# Coordinated interplay between palmitoylation, phosphorylation and SUMOylation regulates kainate receptor surface expression

**DOI:** 10.3389/fnmol.2023.1270849

**Published:** 2023-10-05

**Authors:** Busra P. Yucel, Enaam M. Al Momany, Ashley J. Evans, Richard Seager, Kevin A. Wilkinson, Jeremy M. Henley

**Affiliations:** Centre for Synaptic Plasticity, School of Biochemistry, Biomedical Sciences Building, University of Bristol, Bristol, United Kingdom

**Keywords:** kainate receptors, post-translational modification, palmitoylation, S-acylation, phosphorylation, SUMOylation, membrane trafficking

## Abstract

Kainate receptors (KARs) are key regulators of neuronal excitability and synaptic transmission. KAR surface expression is tightly controlled in part by post-translational modifications (PTMs) of the GluK2 subunit. We have shown previously that agonist activation of GluK2-containing KARs leads to phosphorylation of GluK2 at S868, which promotes subsequent SUMOylation at K886 and receptor endocytosis. Furthermore, GluK2 has been shown to be palmitoylated. However, how the interplay between palmitoylation, phosphorylation and SUMOylation orchestrate KAR trafficking remains unclear. Here, we used a library of site-specific GluK2 mutants to investigate the interrelationship between GluK2 PTMs, and their impact on KAR surface expression. We show that GluK2 is basally palmitoylated and that this is decreased by kainate (KA) stimulation. Moreover, a non-palmitoylatable GluK2 mutant (C858/C871A) shows enhanced S868 phosphorylation and K886 SUMOylation under basal conditions and is insensitive to KA-induced internalisation. These results indicate that GluK2 palmitoylation contributes to stabilising KAR surface expression and that dynamic depalmitoylation promotes downstream phosphorylation and SUMOylation to mediate activity-dependent KAR endocytosis.

## Introduction

Kainate receptors (KARs) are ionotropic glutamate receptors distributed throughout the brain at pre-, post-, and extrasynaptic sites, depending on the neuronal subtype and synapse in question. Compared to AMPARs and NMDARs, ionotropic KAR-mediated synaptic responses are highly restricted to subsets of excitatory synapses but, in addition to being ligand-gated ion channels, KARs also signal via metabotropic, G protein-coupled pathways (Contractor et al., 2011; Lerma and Marques, 2013; Evans et al., 2019). Through this combination of signalling modes, KARs play key modulatory roles in neurotransmitter release, contribute to postsynaptic depolarisation, and play wider regulatory roles in a range of processes including synaptic plasticity, the formation and maintenance of neural circuits, and excitotoxicity and neuronal cell death (Henley et al., 2014; Evans et al., 2019). Unsurprisingly, therefore, KAR dysregulation and/or dysfunction is strongly implicated in multiple neurological and neurodegenerative disorders (Matute, 2011; Fritsch et al., 2014; Henley et al., 2014; Barthet et al., 2022).

KARs are tetrameric assemblies of GluK1-5 core subunits with GluK2/GluK5 heteromers being the most abundant postsynaptic KAR subunit combination (Petralia et al., 1994). KAR trafficking and functional surface expression are tightly regulated through a variety of protein interactions and post-translational modifications (González-González et al., 2012; Lerma and Marques, 2013; Carta et al., 2014; Evans et al., 2017). In particular, the GluK2 subunit is post-translationally modified by phosphorylation (Nasu-Nishimura et al., 2010; Konopacki et al., 2011; Chamberlain et al., 2012), ubiquitination (Salinas et al., 2006; Maraschi et al., 2014), SUMOylation (Martin et al., 2007a) and palmitoylation (Pickering et al., 1995; Copits and Swanson, 2013) in its intracellular C-terminus.

SUMO1 is a 97-residue, 11 kDa protein that is covalently conjugated to specific lysine residues on target proteins to modify substrate function (Wilkinson and Henley, 2010; Henley et al., 2014). GluK2 was the first of many synaptic membrane proteins shown to be SUMOylated (Martin et al., 2007a). Subsequent studies have demonstrated that SUMOylation is involved in multiple neuronal signalling cascades and is strongly implicated in many neurological and neurodegenerative diseases (Henley et al., 2021). SUMOylation of GluK2 at K886 is required for agonist-induced internalisation of surface expressed GluK2-containing KARs and acts to regulate the complement of synaptic KARs (Martin et al., 2007b). Importantly, PKC phosphorylation of S868 enhances GluK2 SUMOylation and is required for KAR LTD at MF-CA3 synapses (Konopacki et al., 2011; Chamberlain et al., 2012; Nair et al., 2021b).

In addition to mediating GluK2 SUMOylation, PKC phosphorylation has been shown to play a variety of roles in GluK2 surface delivery and activity-dependent trafficking, depending on the phosphorylation site and cellular location. For example, ER exit and secretory pathway trafficking of GluK2-containing KARs is restricted by phosphorylation of GluK2 at residues S846 and S868 by PKC (Nasu-Nishimura et al., 2010; Evans et al., 2017). Furthermore, PKC phosphorylation of S846 promotes endocytosis of GluK2-containing KARs, potentially by modulation of the interaction between GluK2 and 4.1 proteins (Copits and Swanson, 2013), and PKC phosphorylation of S868 also participates in recycling of endocytosed GluK2 back to the plasma membrane (Chamberlain et al., 2012). Thus, understanding the context-specific effects of GluK2 phosphorylation, and its interplay with other PTMs, represents an important objective in the field.

Dynamic and reversible S-acylation is the linkage of molecules via thioester bonds. Palmitoylation, a prominent form of cysteine-mediated protein S-acylation, regulates the trafficking, lipid membrane association and surface stability of a wide variety of integral membrane proteins (Daniotti et al., 2017; Jin et al., 2021). Historically, palmitoylation has been difficult to study, so understanding of its targets and functions in neurons remains comparatively limited (Matt et al., 2019). In cell lines, all KAR subunits, except GluK1, can be palmitoylated at two conserved distal cytosolic cysteine residues (C858 and C871 in GluK2; Pickering et al., 1995; Copits and Swanson, 2013). Like phosphorylation and SUMOylation, palmitoylation is a highly dynamic, reversible post-translational process that can enhance the hydrophobicity of proteins to control target protein membrane association and clustering (Hayashi, 2021a,b), especially at lipid rafts, in addition to regulating trafficking and protein–protein interactions (Fukata and Fukata, 2010; Levental et al., 2010). Importantly, however, palmitoylation of GluK2 has not been demonstrated in neurons, and how interplay between palmitoylation, phosphorylation and SUMOylation coordinates KAR surface expression and activity-dependent endocytosis has not been investigated.

Here, we optimised an APEGS palmitoylation assay to investigate the interplay between palmitoylation, phosphorylation and SUMOylation of GluK2. We demonstrate that GluK2 is endogenously palmitoylated in neurons, and that GluK2 palmitoylation is reduced by agonist stimulation. Furthermore, using a non-palmitoylatable GluK2 mutant, we demonstrate that preventing GluK2 palmitoylation enhances PKC-mediated phosphorylation at S868, and SUMOylation at K886, and results in reduced KAR surface expression. Moreover, non-palmitoylatable GluK2 was insensitive to kainate (KA)-induced internalisation. Thus, our data support a model whereby agonist-induced depalmitoylation leads to PKC-mediated phosphorylation and subsequent SUMOylation of GluK2, to promote KAR endocytosis.

## Materials and methods

### Drugs

**Table 1:** 

Drugs	Concentration (μM)	Diluted in	Supplier	Catalog number
GYKI 53655	40	ddH_2_O	Tocris	2555
KA	10	ddH_2_O	Tocris	0222
TTX	2	ddH_2_O	Tocris	1069

### Primary antibodies

**Table 2:** 

Antibody	Species	Supplier	Catalog number	Dilution
GluK2 (WB)	Rabbit	Millipore	04–921	1:5000
Myc (WB)	Rabbit	Cell signalling	71D10	1:2000
Flag (WB)	Mouse	Sigma	F3165	1:1000
GFP (ICC)	Chicken	Abcam	ab13970	1:1000
GFP (WB)	Rat	Chromotek	3H9-100	1:2500
β-actin (WB)	Mouse	Sigma	A5441	1:10000

### Neuronal cell cultures

Hippocampal cultures derived from E18 Han Wistar rats were prepared as described previously (Konopacki et al., 2011). The cortical cells were plated in 6-well dishes (500 k per well) for biochemistry and hippocampal cells were plated on coverslips (100 k per coverslip) for imaging. Cells were incubated for a period of 19–21 days before use. Initially, the neuronal plating media consisted of Neurobasal (Gibco) supplemented with 10% Horse Serum, 2% B27, 2 mM Glutamax, and Penicillin–Streptomycin. After 2 h, the media was switched to feeding media lacking horse serum.

### Transduction with lentivirus

To conduct GluK2 knockdown experiments, shRNA sequences specifically targeting GluK2 were inserted into a modified pXLG3-GFP vector (Wilkinson et al., 2022), under the control of an H1 promoter. The shRNA target sequences used were as follows:

Control, non-targeting shRNA (SCR): AATTCTCCGAACGTGTCAC

GluK2-targeting shRNA (SH1): GCCGTTTATGACACTTGGA

For knockdown-replacement experiments, N-terminally YFP-myc-tagged rat GluK2 (WT or indicated mutants) containing five silent point mutations rendering them insensitive to the GluK2 shRNA were cloned into the pXLG3-GluK2 shRNA plasmid, in place of the GFP. The viral particles containing these constructs were generated in HEK293T cells as described previously (Wilkinson et al., 2022). Subsequently, the harvested viral particles were added to cortical neurons at 12–14 days *in vitro* (DIV) and allowed to incubate for 7 days before being used for further experiments.

### Sustained KA stimulation

Rat cortical neurons were plated in a 6-well dish with a density of 500,000 cells per well. The cells were incubated at 37°C until reaching a developmental stage of DIV 19–21 DIV. On the experiment day, the cells were subjected to pre-treatment by the addition of 2 μM tetrodotoxin (TTX, a Na^2+^ channel blocker; Tocris) and 40 μM GYKI 53655 (a selective AMPAR blocker; Tocris) to the culture medium for 30 min. Following pre-treatment, KA incubation (10 μM for 20 min at 37°C) was performed in the continued presence of TTX and GYKI 53655. Control cells were treated with a vehicle instead of KA.

### Neuronal surface biotinylation

Following KA treatment, surface proteins on the cell were labelled using membrane impermeant Sulfo-NHS-SS-Biotin (ThermoFisher). The biotinylation process was performed on ice and using ice-cold Earle’s Buffer following a detailed protocol we have published previously (Nair et al., 2021a). An aliquot (10% of volume) of the biotinylated lysate was transferred to a new Eppendorf and the remaining 90% was incubated with streptavidin coupled beads, washed extensively, and pulled down to isolate biotinylated surface proteins. Following elution from the streptavidin beads half of the eluent was run on SDS–PAGE and Western blotted with anti-myc and anti-β-actin antibodies.

### Transfection of HEK293T cells

Human Embryonic Kidney 293 T (HEK293T) cells were cultured in high-glucose DMEM containing glutamine (Sigma) supplemented with 10% FBS and 2% Penicillin–Streptomycin. HEK293T cells were plated for transfection in 60 mm dishes the day before transfection. 2.5 μg for each DNA were added to 500 μL plain DMEM, followed by the addition of Lipofectamine (1.5 μL per μg DNA), vortexed briefly, and incubated for 20 min. The media on the cells was then aspirated and replaced with DMEM containing 10% FBS, and the DNA-Lipofectamine mixture was added dropwise to the cells, and the transfected cells were incubated for 48 h before use.

### Detection of post-translational modifications

HEK293T cells or neurons were transfected or infected, respectively, with wild-type or point mutants of YFP-myc-tagged GluK2 (non-palmitoylatable, C858A/C871A; non-SUMOylatable, K886R; phospho-null, S846A/S868A; phospho-mimetic, S846D/S868D; Non-palmitoylated, non-phosphorylated single mutants, C858A/C871A/S846A and C858A/C871A/S868A).

#### SUMOylation

HEK293T cells were transfected with GFP or YFP-myc-GluK2 constructs and Flag-SUMO1 and Flag-Ubc9. 48 h after transfection, cells were stimulated with 100 μM KA and lysed in lysis buffer (20 mM Tris–HCl (pH 7.4), 137 mM NaCl, 2 mM sodium pyrophosphate, 2 mM EDTA, 1% Triton-X 100, 0.1% SDS, 25 mM β-glycerophosphate, 10% glycerol, 20 mM N-ethylmaleimide (NEM, freshly prepared), and 1x complete protease inhibitors). Lysates were sonicated briefly and incubated on ice for 20 min, before being cleared by centrifugation at 16000 g for 20 min at 4°C. The supernatant was then taken, and GluK2 was pulled down using GFP-Trap A beads (ChromoTek) overnight at 4°C. After three washes in the lysis buffer, bound proteins were detected by SDS–PAGE followed by Western blotting using anti-Flag and anti-GFP antibodies.

#### Acyl-PEGyl Exchange Gel Shift (APEGS) assay

We combined two previously published protocols to optimise the APEGS assay for GluK2 (Percher et al., 2016; Yokoi et al., 2016; Kanadome et al., 2019). Briefly, the APEGS assay comprises four steps: (1) disruption of disulphide bonds; (2) blocking free cysteine residues; (3) cleaving palmitoyl-thioester linkages with hydroxylamine; (4) labelling previously palmitoylated but now newly exposed cysteine residues with mPEG-MAL-10 k. This results in a ~ 10 kDa increase in mass for each palmitoylated cysteine on SDS–PAGE (Yokoi et al., 2016).

Neurons were homogenised in PBS (Gibco) containing 4% SDS, 5 mM EDTA, and protease inhibitors (cOmplete). After sonication, supernatant proteins (2 mg/mL) were reduced with 25 mM TCEP (ThermoFisher) for 1 h at 55°C, followed by alkylation of free cysteine residues with 50 mM NEM (Sigma) overnight at RT. The samples were then subjected to methanol/chloroform precipitation and the precipitate spun down, washed, and the protein pellet resuspended and incubated in 2 M hydroxylamine (NH_2_OH) (Sigma), pH 7.0, 5 mM EDTA, 0.2% (w/v) Triton X-100 for 1 h at 37°C to cleave palmitoylation thioester bonds. After a second methanol/chloroform precipitation samples were incubated with 7 mM mPEG-MAL-10 k (Sigma) for 2 h at RT to label newly exposed cysteinyl thiols. As a negative control, mPEG-MAL-10 k was omitted. Following a third methanol/chloroform precipitation the protein pellet was resuspended with SDS-sample buffer (without β-ME), and samples were left in the water bath for 10 min. 2% β-ME was added to each sample and samples were boiled at 95°C for 3 min. The samples were run on a 6% SDS–PAGE gel, transferred to a membrane for 120 min and blotted with anti-myc or anti-GluK2 antibodies.

#### Phos-tag gel electrophoresis

50 μM 6% phos-tag gels (Fujifilm) were prepared following the manufacturer’s protocol. Cells were lysed in 200 μL 2x sample buffer, sonicated and 20 μL of each sample run on phos-tag gel. Gels were washed with 10 mM EDTA in dH_2_O for 10 min followed by Western blotting. Blots were blocked with milk and myc (Rb) antibody was used to detect GluK2 bands.

#### Quantitative Western blots

To quantify the level of GluK2 SUMOylation, phosphorylation, and palmitoylation the ratio of SUMOylated to non-SUMOylated, phosphorylated to non-phosphorylated and palmitoylated to non-palmitoylated GluK2 was determined.

For palmitoylation, the signals from unconjugated, single, and double palmitoylated bands were summated, averaged, and normalised to 100.

### Live surface labelling

Hippocampal neurons on 25 mm coverslips were transfected with YFP-myc-GluK2 (WT or C2A) at DIV 9–10. At DIV 14–15, media was replaced with pre-warmed HBSS containing 2 μM TTX and 40 μM GYKI53655 for 30 min to block neuronal activity and AMPARs, respectively. Coverslips were then incubated with 10 μM KA in the same media or vehicle control for 20 min. After KA stimulation, neurons were incubated with chicken anti-GFP antibody (ab13970, 1:1000) in media for 20 min at 4°C. Next, neurons were washed quickly 5x in cold PBS before being fixed with pre-warmed (37°C) 4% PFA (ThermoFisher) for 20 min. Following fixation, neurons were washed 3x with PBS and then treated with glycine (100 mM in PBS, Severn Biotech) for 1 min after which they were washed 3x in PBS. Then, 3% BSA was used for blocking for 10 min before the incubation with Alexa 647-conjugated anti-chicken secondary antibody (1:400) in 3% BSA for 1 h at room temperature in darkness. Neurons were then washed 3x with PBS and then permeabilised using 3% BSA and 0.1% Triton-X 100 (Fisher Scientific) for 10 min in darkness. After that, coverslips were incubated with chicken anti-GFP (1:1000) again to label the total transfected GluK2 for 1 h in darkness. Neurons were washed with PBS (3x) before the addition of Cy2-conjugated anti-chicken secondary antibody (1:400, green) in darkness for 1 h. Coverslips were then washed 3x before being mounted on prelabelled glass slides using Fluoromount-G with DAPI (eBioscience). Glass slides were kept in darkness to dry out for 24–48 h before being imaged.

### Imaging analysis

Confocal imaging was performed using a Leica SP5-AOBS confocal laser scanning microscope linked to a Leica DMI 6000 inverted epifluorescence microscope with the laser lines: 405 (blue for the nucleus), 488 (green for the total), 633 (far red for the surface). The transfected neurons were imaged by looking for the total labelling of GluK2 (green cells). A 63x oil immersion lens was used for image acquisition. Each image is composed of 5–6 stacks (0.4–0.5 μm stack interval) that were projected by maximum intensity. The untreated WT GluK2 condition was used to optimise the settings, which were kept constant throughout the same experiment. The immunofluorescence was quantified using ImageJ (FIJI).

### Statistical analysis

All graphs and statistics were generated on GraphPad Prism version 9.0. The details of the statistical tests performed on each experiment are explained in the figure legend along with *p*-values and error bars.

## Results

### GluK2 can be palmitoylated at C858 and C871 in HEK293T cells

**Figure 1:**
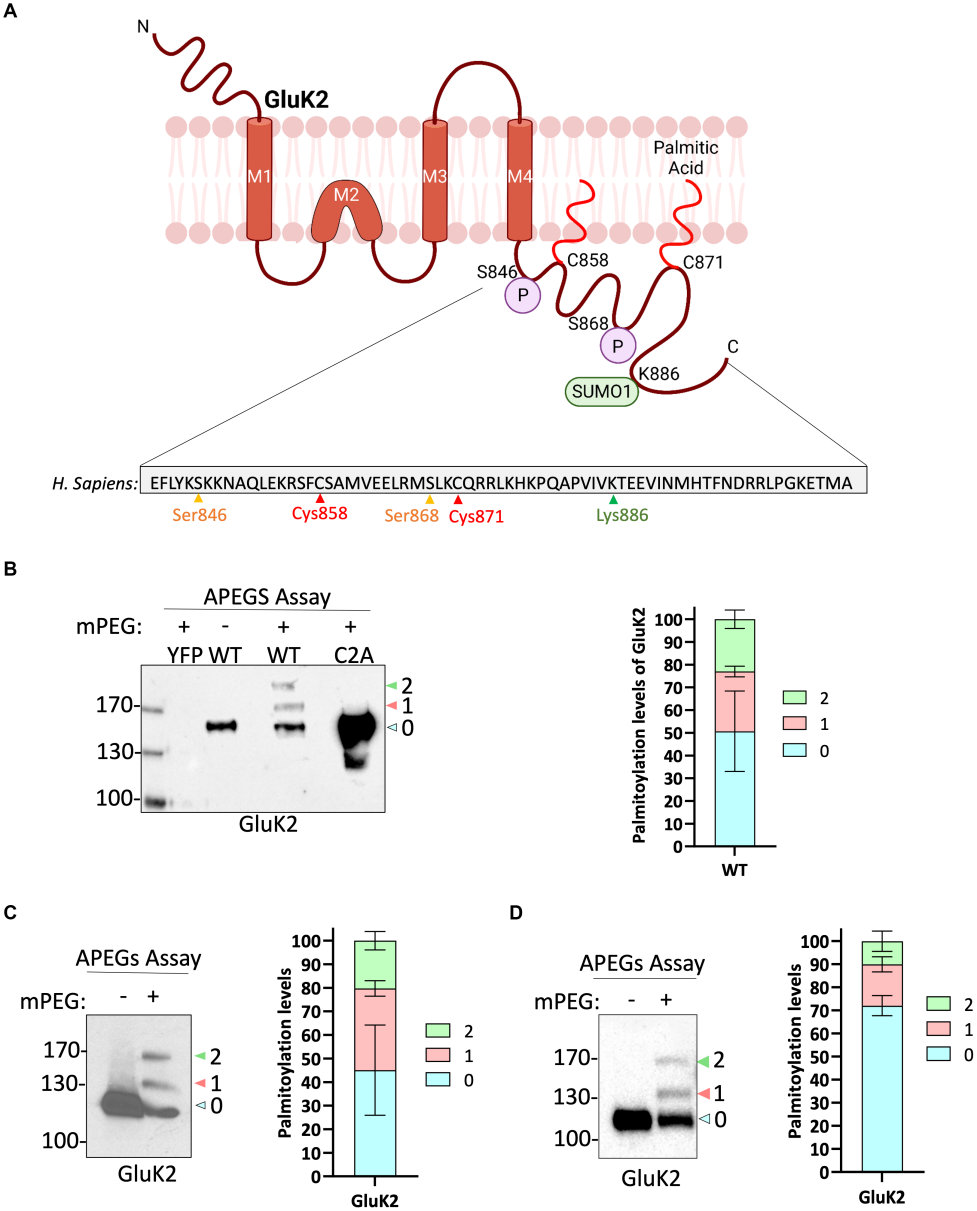
GluK2 is palmitoylated in HEK293T cells, mouse brain and cultured rat cortical neurons. **(A)** The GluK2 subunit of KARs undergoes a complex array of post-translational modifications in its C-terminus, which include palmitoylation at cysteine residues 858 and 871, phosphorylation at serine residues 846 and 868, and SUMOylation at lysine residue 886. **(B)** HEK293T cells were transfected with YFP, YFP-myc-GluK2 (WT), or the non-palmitoylated form of YFP-myc-GluK2 (C2A). An APEGS assay was then performed to evaluate the palmitoylation levels of recombinant homomeric GluK2. The results of this assay are reflected by a band shift, as a result of the addition of 10 kDa mPEG-MAL-10 k molecules to the exposed cysteine residues. Data from the blot analysis indicates that GluK2 is palmitoylated at two distinct cysteine residues (C858/C871) since the double cysteine mutant did not exhibit any palmitoylation. In recombinant systems, approximately 50% of the total GluK2 protein exhibits palmitoylation at one or both of its cysteine residues. 27% of total GluK2 exhibits palmitoylation at a single cysteine residue, whilst an additional 23% of GluK2 was palmitoylated at both cysteine residues. In the analysis, “0” indicates non-palmitoylated GluK2, whilst “1” represents GluK2 that is palmitoylated at a single cysteine residue and “2” signifies GluK2 that is palmitoylated at both cysteine residues. *N* = 3 separate experiments. Error bars = SEM. **(C)** A mouse cortex was used as a source of lysate for the APEGS assay. The negative control lane (-mPEG-MAL-10 k) showed no evidence of palmitoylation, whilst endogenous GluK2 was observed to exist in two palmitoylated forms. The graph depicts the palmitoylation states of native GluK2, with 55% of the total population exhibiting palmitoylation. Approximately 20% of the total GluK2 is palmitoylated at a single cysteine residue, whilst 35% is palmitoylated at both cysteine residues. In the analysis, “0” indicates non-palmitoylated GluK2, whilst “1” represents GluK2 that is palmitoylated at a single cysteine residue and “2” signifies GluK2 that is palmitoylated at two cysteine residues. *N* = 3 separate experiments. Error bars = SEM. **(D)** Rat cortical cells were plated and used for APEGS assay at DIV 19–21. 31% of total GluK2 was palmitoylated at either single (14.5%) or double (16.5%) cysteines. In the analysis, “0” indicates non-palmitoylated GluK2, whilst “1” represents GluK2 that is palmitoylated at a single cysteine residue and “2” signifies GluK2 that is palmitoylated at two cysteine residues. *N* = 3 separate dissections. Error bars = SEM.

The topology of GluK2, the amino acid sequence of the intracellular C-terminal domain and sites of key post-translational modifications are illustrated in Figure 1A. To measure GluK2 palmitoylation we modified the Acyl-PEGyl Exchange Gel Shift (APEGS) assay (Kanadome et al., 2019; Speca and Diaz, 2020). In this assay, cells are lysed in the presence of a reducing agent to break disulphide bonds, followed by treatment with the alkylating agent *N-*ethylmaleimide to ‘block’ thiol groups on free cysteines. Palmitoyl moieties are then cleaved from modified cysteines using hydroxylamine, and lysates treated with a high-molecular weight thiol-reactive mPEG-MAL-10 K to specifically label formerly palmitoylated cysteines. Proteins modified by palmitoylation therefore exhibit a mass shift of ~10 kDa when analysed by Western blotting.

We first validated the APEGS assay by expressing YFP-myc-GluK2 wild-type (GluK2-WT) or a double cysteine mutant C858/C871A (GluK2-C2A), which has previously been reported to be non-palmitoylatable in cell lines (Pickering et al., 1995; Copits and Swanson, 2013), in HEK293T cells. As shown in Figure 1B, GluK2-WT showed two higher molecular weight bands corresponding to singly and doubly palmitoylated GluK2. These bands were absent in the negative control lane in which mPEG-MAL-10 K was omitted, confirming them as genuine mPEG-MAL-10 k adducts. Consistent with previous reports (Pickering et al., 1995; Copits and Swanson, 2013), no higher molecular weight bands were present in the GluK2-C2A mutant, even though the expression of the latter construct was much greater, confirming C858 and C871 as the only palmitoylated cysteines in GluK2. Quantification of our data shows that ~50% of the GluK2 expressed in HEK293T cells is not palmitoylated, whereas ~27% is singly palmitoylated and ~ 23% is doubly palmitoylated (Figure 1B).

### Endogenous GluK2 is palmitoylated in neurons

Having established that recombinant GluK2 is palmitoylated in HEK293T cells we next used the APEGS assay to investigate palmitoylation of endogenous GluK2 in mouse cortex (Figure 1C) and cultured rat cortical neurons (Figure 1D). Consistent with the HEK293T cell data, in both systems we detected two palmitoylated forms of endogenous GluK2. In mouse cortex, ~35% of endogenous GluK2 is singly and ~ 20% doubly palmitoylated, whilst in DIV 19–21 rat cortical neurons ~14.5% was singly and ~ 16.5% doubly palmitoylated. Together, these data confirm the palmitoylation of endogenous GluK2.

### KA stimulation decreases GluK2 palmitoylation

**Figure 2:**
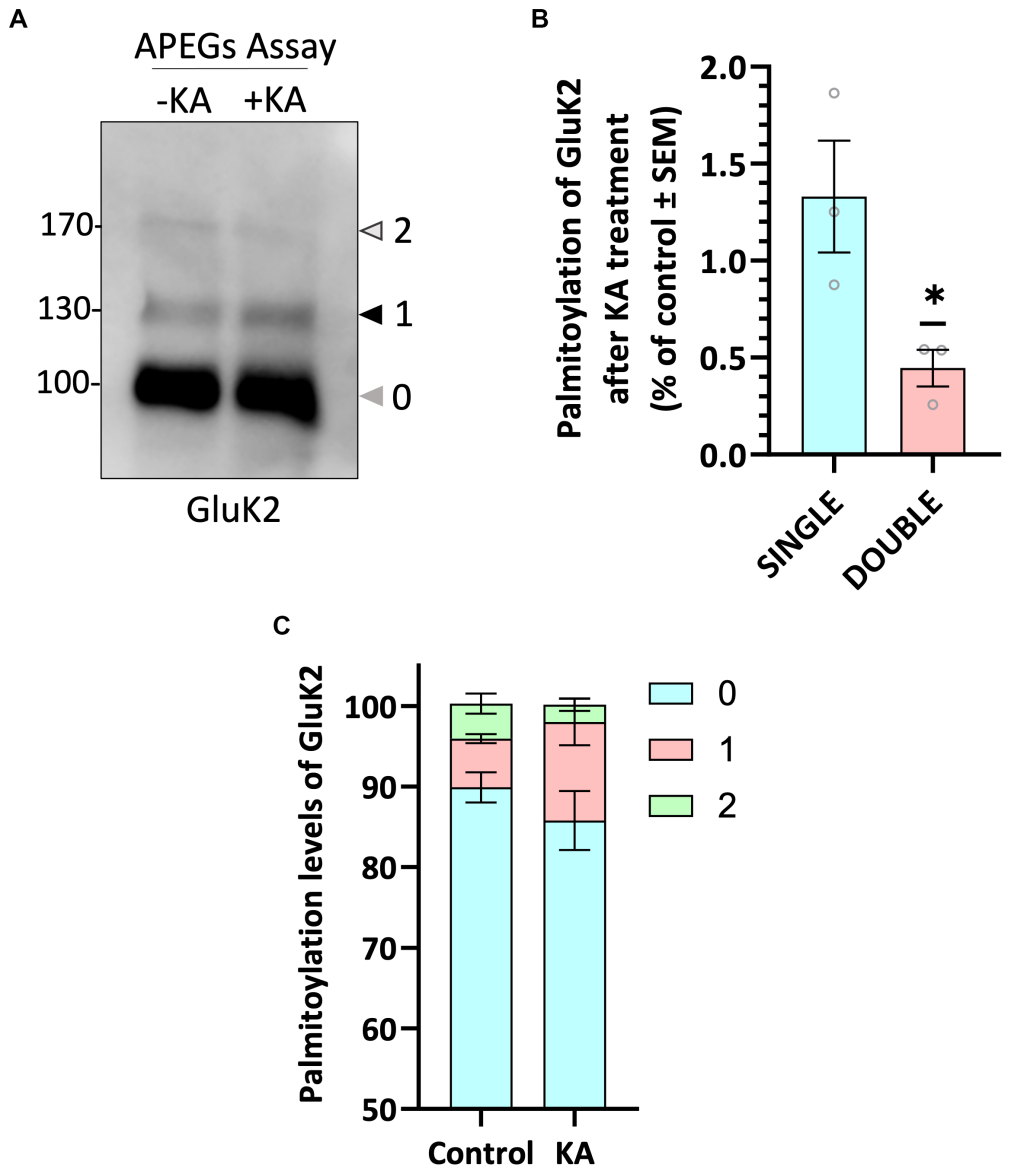
GluK2 palmitoylation decreases with KA stimulation. **(A)** DIV 19–21 cultured cortical neurons were pre-incubated with TTX and GYKI53655 followed by a stimulation with 20 μM KA for 5 min. Lysates were subjected to the APEGS assay. **(B)** The graph shows the comparison of single and double palmitoylation in control and KA-induced cells. There was a significant decrease in the level of double palmitoylated GluK2 in KA-induced neurons compared to the control. *N* = 3 independent dissections. One sample *t*-test (*p < 0.05). Error bars = SEM. **(C)** The graph depicts the palmitoylation states of native GluK2, with 10% of the total population exhibiting palmitoylation. Approximately 5% of the total GluK2 is palmitoylated at a single cysteine residue, whilst 5% is palmitoylated at both cysteine residues in control, whereas nearly 15% of total GluK2 is palmitoylated with 12% in single cysteine and 3% in double cysteines in KA stimulated neurons. In the analysis, “0” indicates non-palmitoylated GluK2, whilst “1” represents GluK2 that is palmitoylated at a single cysteine residue and “2” signifies GluK2 that is palmitoylated at two cysteine residues. *N* = 3 separate experiments. Error bars = SEM.

We next wondered whether palmitoylation of endogenous GluK2 was dynamically regulated by KAR activation. We therefore pre-incubated neurons with 2 μM TTX and 40 μM GYKI 53655, to block spontaneous activity and prevent KA-induced activation of AMPARs, respectively, and then stimulated neurons with 20 μM KA for 5 min. Interestingly, KAR activation led to a decrease in GluK2 double palmitoylation (Figures 2A–C), suggesting that agonist-stimulation leads to a rapid depalmitoylation of GluK2.

### GluK2 is palmitoylated at C858 and C871 in cultured neurons

**Figure 3:**
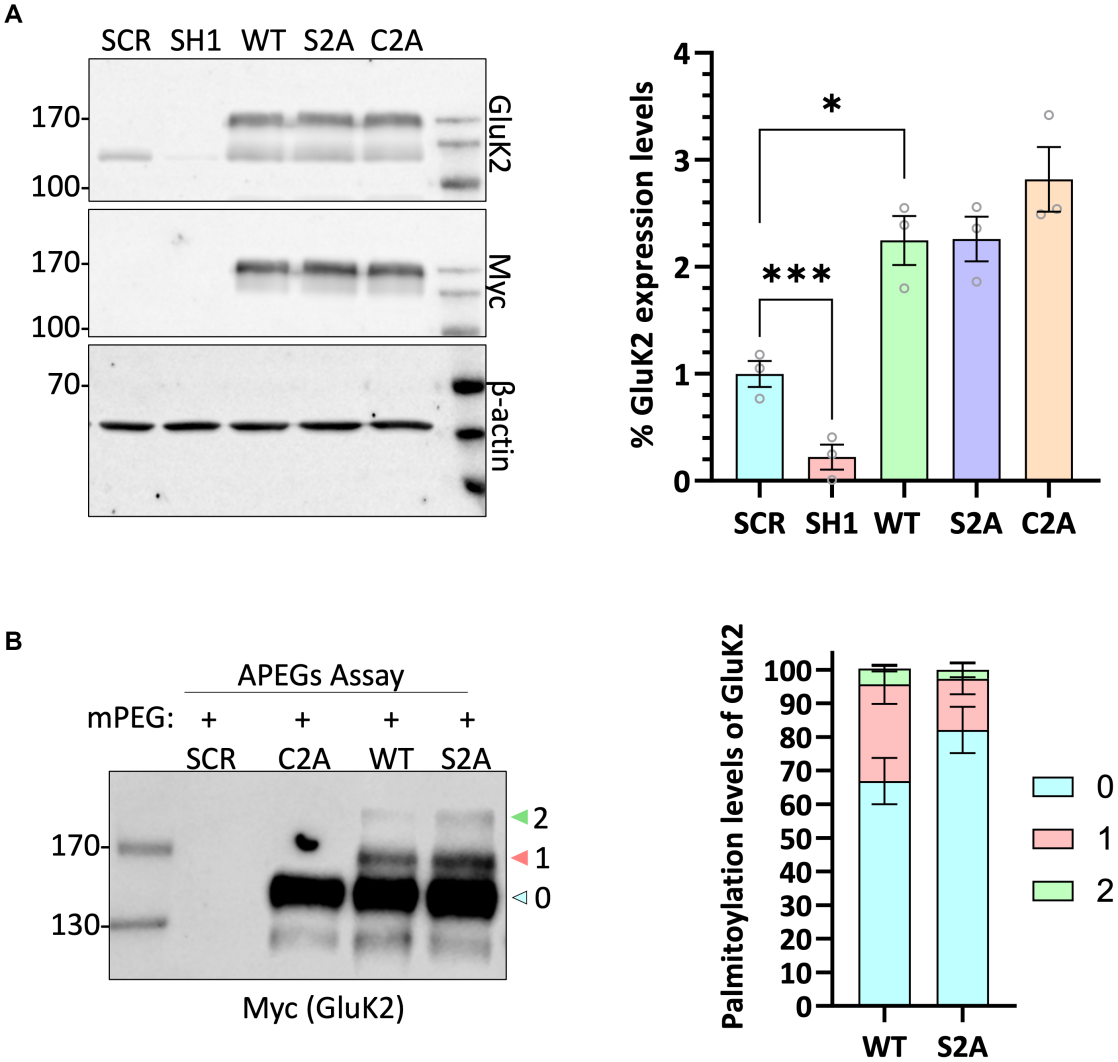
GluK2 is palmitoylated at C858 and C871 in cultured cortical neurons. **(A)** DIV 14 rat cortical neurons were infected with Scrambled control, GluK2 knockdown, or GluK2 knockdown-replacement lentiviruses. SCR = scrambled control; SH1 = GluK2 knockdown; WT = GluK2 knockdown and replacement with YFP-myc-tagged wild-type GluK2; S2A = GluK2 knockdown and replacement with non-phosphorylatable YFP-myc-GluK2-S2A; C2A = GluK2 knockdown and replacement with non-palmitoylatable YFP-myc-GluK2-C2A. The blots were probed with myc, GluK2 and β-actin antibodies. The expression levels of the different GluK2 mutants were normalised to β-actin and then expressed as a proportion of the SCR control value. One-way ANOVA with Dunnett’s *post-hoc* test was applied (*p < 0.05, ***p < 0.001). *N* = 3 dissections. Error bars = SEM. **(B)** Conditions as for A followed by APEGS analysis of GluK2 WT, C2A and S2A palmitoylation. The results confirm that the double cysteine GluK2 mutant, C858A/C871A is not palmitoylated in neurons. The bar chart shows ~30% GluK2 WT is palmitoylated with ~26% palmitoylated at a single cysteine and ~ 4% palmitoylated at both cysteines. ~20% GluK2 S2A is palmitoylated with ~18% palmitoylated at a single cysteine and ~ 2% palmitoylated at both cysteines. “0” indicates non-palmitoylated GluK2, “1” indicates palmitoylation at a single cysteine and “2” indicates palmitoylated at two cysteines.

To further investigate GluK2 palmitoylation and its interplay with other post-translational modifications, we used a lentiviral GluK2 shRNA knockdown (KD) and replacement strategy in rat cortical neuronal cultures (Wilkinson et al., 2022). We engineered lentiviral constructs that knocked down endogenous GluK2 and simultaneously expressed shRNA-insensitive YFP-myc-tagged GluK2 variants. Specifically, we replaced endogenous GluK2 with either GluK2-WT, non-palmitoylatable GluK2-C2A or a phosphorylation-deficient GluK2-S2A (S846/868A), which lacks two previously characterised PKC phosphorylation sites in the GluK2 C-terminus (Nasu-Nishimura et al., 2010). Neurons were infected with KD-replacement lentivirus at DIV 14 and after a further 7 days cells were lysed and Western blotted to ensure that each YFP-myc-GluK2 mutant was expressed at similar levels and the expected molecular weight (~150 kDa; Figure 3A).

We then assessed the palmitoylation of GluK2-WT, GluK2-C2A and GluK2-S2A using the APEGs assay. As expected, GluK2-WT showed bands corresponding to single and double palmitoylation, but these bands were absent for GluK2-C2A, confirming in neurons that only C858 and C871 can be palmitoylated. Consistent with the overall levels of palmitoylation observed for endogenous GluK2 in cultured neurons, ~30% of total YFP-myc-tagged GluK2-WT was palmitoylated with ~26% palmitoylated at a single cysteine and ~ 4% palmitoylated at both cysteines whereas ~20% GluK2 S2A is palmitoylated with ~18% palmitoylated at a single cysteine and ~ 2% palmitoylated at both cysteines (Figure 3B).

### Non-palmitoylatable GluK2 shows enhanced PKC phosphorylation at S868

We have shown previously that agonist stimulation of GluK2 promotes PKC-mediated phosphorylation at S868, leading to enhanced SUMOylation at K886 and receptor endocytosis (Martin et al., 2007a; Konopacki et al., 2011; Chamberlain et al., 2012). Using recombinant proteins it has also been reported previously that non-palmitoylatable GluK2 is a better substrate for phosphorylation by PKC than WT GluK2 (Pickering et al., 1995). Since KA stimulation leads to a reduction in GluK2 palmitoylation, we wondered whether GluK2 depalmitoylation promotes PKC phosphorylation and subsequent KAR endocytosis. To examine this directly, we used phos-tag gel electrophoresis (Nagy et al., 2018; O'Donoghue and Smolenski, 2022) to analyse the phosphorylation of non-palmitoylatable GluK2.

**Figure 4:**
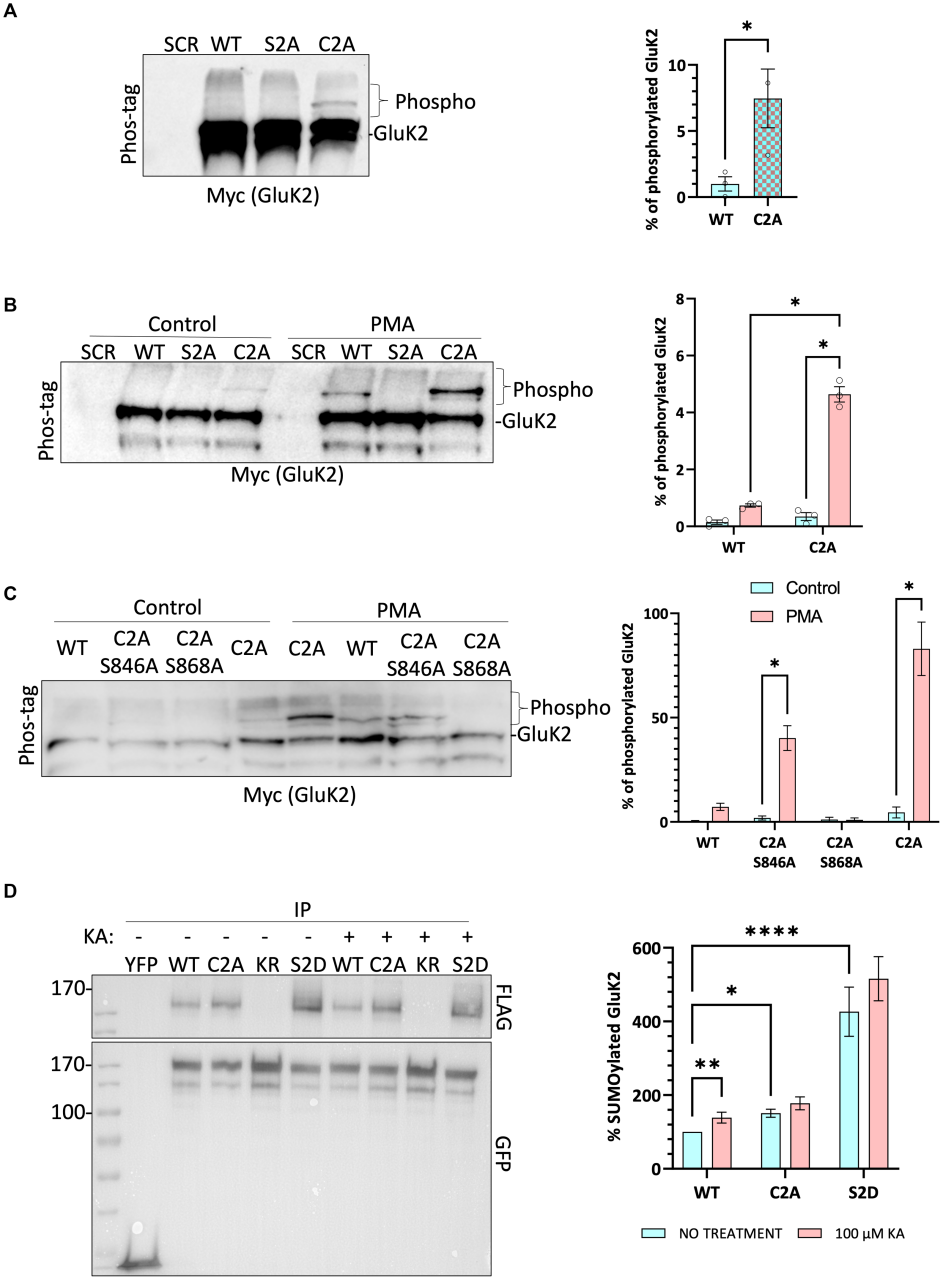
Non-palmitoylated GluK2 is a better substrate for protein kinase C than wild-type GluK2. **(A)** On DIV 14, cultured rat cortical neurons were infected with the SCR control lentivirus or GluK2 knockdown-replacement lentiviruses that mediate simultaneous knockdown and expression of the replacement protein: SCR = scrambled control; WT = GluK2 knockdown and replacement with YFP-myc-tagged wild-type GluK2; S2A = GluK2 knockdown and replacement with non-phosphorylatable YFP-myc-GluK2-S2A; C2A = GluK2 knockdown and replacement with non-palmitoylatable YFP-myc-GluK2-C2A. After 7 days of incubation, cells were lysed with 2x sample buffer and run on a phos-tag gel followed by Western blotting for myc. Band shifts indicate GluK2 phosphorylation for the C2A mutant, but not WT or S2A. Phosphorylated GluK2 was first normalised to non-phosphorylated GluK2 and C2A expressed as a proportion of the WT. Student *t*-test (*p < 0.05). **(B)** As for A except that after 7 days of incubation cells were incubated with 1 μM PMA for 20 min and lysed with 2x sample buffer, sonicated, and run on a phos-tag gel. Western blots were then probed for myc. Phosphorylated GluK2 bands in WT and C2A were first normalised to unconjugated GluK2. Two-way ANOVA with Tukey’s multiple comparisons test. WT vs. mutant, degrees of freedom (df) = 1, *F* = 10.39. Untreated vs. treated, df = 1, *F* = 14.71. (****p < 0.0001). *N* = 3 independent dissections. Error bars = SEM. **(C)** Analysis of individual PKC phosphorylation sites. As for B, except neurons were infected at DIV 14 with SCR, or knockdown-replacement lentiviruses: WT = YFP-myc-GluK2; C2A-S846A or C2A-S868A = single PKC phosphorylation-deficient and non-palmitoylatable; C2A = non-palmitoylatable YFP-myc-GluK2. After 7 days of incubation, cells were incubated with 1 μM PMA for 20 min and lysed with 2x sample buffer, sonicated, and run on a phos-tag gel. Western blot was probed for myc. Two-way ANOVA with Tukey’s multiple comparisons test. WT vs. mutants, df = 3, *F* = 6.261. Untreated vs. treated, df = 1, *F* = 15.39 (*p < 0.05). *N* = 3 independent dissections. Error bars = SEM. **(D)** Preventing palmitoylation of GluK2 mimics agonist-induced SUMOylation. HEK293T cells were transfected with either YFP or the indicated YFP-myc-GluK2 constructs (as above plus KR = non-SUMOylatable mutant and S2D = double PKC phosphomimetic mutant), along with FLAG-tagged SUMO1 and FLAG-tagged Ubc9. 48 h post-transfection, cells were stimulated with 100 μM KA (20 min). GluK2 was immunoprecipitated (IP) using GFP-trap and the levels of SUMOylated GluK2 were detected in the different conditions by Western blotting using an anti-FLAG antibody. SUMOylated GluK2 signal was first normalised to the amount of YFP-GluK2 pulled down before being normalised to the untreated WT. Two-way ANOVA with Tukey’s multiple comparisons test. WT vs. mutants, df = 2, *F* = 282.4. Untreated vs. treated, df = 1, *F* = 15.50. (*p < 0.05, **p < 0.01, ****p < 0.0001). *N* = 6 separate experiments. Error Bars = SEM.

Endogenous GluK2 was knocked down in cortical neurons and replaced with YFP-myc-tagged GluK2-WT, non-palmitoylatable GluK2-C2A or, as a negative control for PKC phosphorylation, GluK2-S2A (S846/868A). Neurons were infected with the relevant lentivirus at DIV 14 and lysed at DIV 21 and samples were subjected to phos-tag gel electrophoresis and Western blotting. Under basal conditions no band shifts indicating phosphorylated GluK2 were detected in GluK2-WT or PKC phospho-null GluK2-S2A. Interestingly, however, we observed clear phosphorylation of the non-palmitoylatable GluK2-C2A mutant (Figure 4A), suggesting this mutant may be more phosphorylated than its WT counterpart.

To investigate the effect of GluK2 palmitoylation on PKC phosphorylation neurons were incubated with the PKC activator phorbol 12-myristate 13-acetate (PMA; 1 μM for 20 min) before lysis and subjected to phos-tag gel electrophoresis followed by Western blotting (Figure 4B). As expected, no PKC phosphorylation was detected for GluK2-S2A, but both GluK2-WT and GluK2-C2A showed enhanced phosphorylation in response to PKC activation. Furthermore, the non-palmitoylatable GluK2-C2A was phosphorylated to a ~ 5-fold greater extent than GluK2-WT, confirming that non-palmitoylatable GluK2 is a better substrate for PKC phosphorylation in neurons, and suggesting that agonist-induced depalmitoylation of GluK2 may lead to phosphorylation by PKC.

We next examined which PKC site on GluK2 shows enhanced phosphorylation in the absence of receptor palmitoylation. We generated non-palmitoylatable mutants of GluK2 in which single PKC phosphorylation sites (S846 or S868) were mutated to alanine. Cortical neurons were infected with knockdown-replacement lentiviruses expressing YFP-myc-tagged GluK2-WT, GluK2-C2A, or the non-palmitoylatable single PKC phosphorylation mutants GluK2-C2A/S846A or GluK2-C2A/S868A at DIV 14. After 7 days, cells were incubated with 1 μM PMA for 20 min, subjected to phos-tag gel electrophoresis and blots were probed for YFP-myc-GluK2 (Figure 4C). GluK2-WT, GluK2-C2A and GluK2-C2A/S846A were phosphorylated in response to PKC activation, but GluK2-C2A/S868A was not, suggesting that the enhanced phosphorylation of the non-palmitoylatable GluK2 mutant occurs at S868. Together, these data suggest that in the absence of palmitoylation GluK2 is more readily phosphorylated by PKC at S868.

### Non-palmitoylatable GluK2 exhibits enhanced SUMOylation

Because the non-palmitoylatable mutant of GluK2 exhibits enhanced PKC phosphorylation at S868, and phosphorylation at this site has been demonstrated to enhance GluK2 SUMOylation (Konopacki et al., 2011; Chamberlain et al., 2012), we next examined SUMOylation of the non-palmitoylatable GluK2-C2A. We transfected HEK293T cells with YFP-myc-tagged GluK2-WT, non-palmitoylatable GluK2-C2A, double PKC phospho-mimetic GluK2-S2D or, as a negative control, non-SUMOylatable GluK2-K886R. To aid detection of SUMOylation, cells were also co-transfected with FLAG-SUMO1 and FLAG-Ubc9. 48 h post-transfection, cells were stimulated with 100 μM KA for 20 min before lysis and GluK2 SUMOylation was quantified by GFP-trap pulldown, to isolate YFP-myc-tagged GluK2, followed by blotting for FLAG-SUMO1 (Figure 4D). Under basal conditions there was greater SUMOylation of non-palmitoylatable GluK2-C2A than GluK2-WT, consistent with enhanced phosphorylation of this mutant at S868. Furthermore, whilst KA stimulation evoked a significant increase in SUMOylation of GluK2-WT, SUMOylation of GluK2-C2A was not further enhanced. Taken together, these data suggest that enhanced PKC phosphorylation of non-palmitoylated GluK2 at S868 increases GluK2 SUMOylation and support a model whereby agonist-induced depalmitoylation of GluK2 is required to elicit receptor SUMOylation in response to KA.

### Decreased surface expression of non-palmitoylatable GluK2

**Figure 5:**
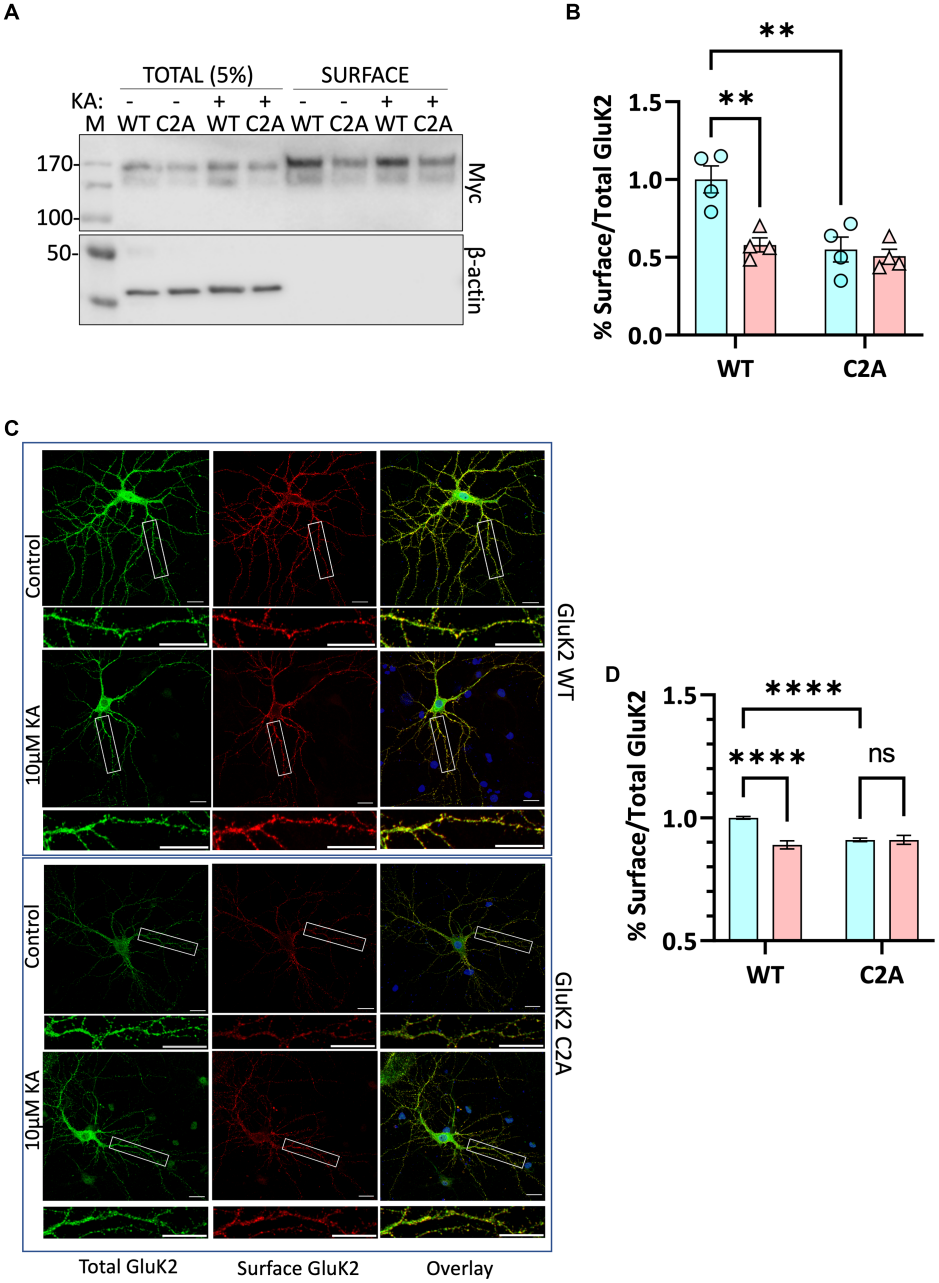
**(A)** Surface labelling experiment confirms that preventing GluK2 palmitoylation reduces surface expression and occludes agonist-induced internalisation. Cultured rat cortical neurons were infected with lentiviruses as indicated: SCR, and knockdown-replacement: YFP-myc-GluK2 (WT) or double non-palmitoylated YFP-myc-GluK2 (C2A) at DIV 14. After 7 days of incubation neurons were pre-treated with 2 μM TTX and 40 μM GYKI53655 for 30 min before being treated with 10 μM KA for 20 min in the same medium. Surface biotinylation experiments were performed followed by streptavidin pulldown and Western blotting. 5% of each cell lysate was run on the gel as Total and half of the proteins eluted from streptavidin beads were run as Surface. **(B)** Quantification of surface biotinylation data shown in panel **(A)**. Surface GluK2 normalised to total levels showing that the KA-evoked decrease in GluK2 WT is occluded in the non-palmitoylatable C2A mutant. Two-way ANOVA with Tukey’s multiple comparisons test (*p < 0.05, **p < 0.01, ***p < 0.001, ****p < 0.0001). *N* = 4 dissections. Error Bars = SEM. **(C)** Confocal imaging confirms that preventing GluK2 palmitoylation reduces surface expression and occludes agonist-induced internalisation. Representative images of hippocampal neurons transfected with YFP-myc-tagged GluK2 (WT or non-palmitoylatable C2A) at DIV 9. At DIV 14 neurons were pre-treated with 2 μM TTX and 40 μM GYKI53655 for 30 min before being treated with 10 μM KA (20 min). Live labelling (using anti-GFP antibody followed by Alexa 647) was used to label the surface expressed GluK2 receptors (red). Total receptors were labelled with anti-GFP followed by Cy2 (green) after staining surface receptors and permeabilization, as described in the methods. 5–15 cells per condition per experiment (a total of 3 independent experiments) were analysed using ImageJ Fiji software. **(D)** Quantification of the fluorescence imaging data shown in panel **(C)**. The results are presented as the surface/total ratio of GluK2 expressed as a ratio of the untreated WT in the dendrites. Data presented as a percentage of the untreated WT. Two-way ANOVA with Tukey’s multiple comparisons test (*p < 0.05, **p < 0.01, ***p < 0.001, ****p < 0.0001). Scale bars: 20 μm (main panel) and 5 μm (magnification panel). 5–15 cells/condition/experiment. *N* = 3 independent experiments. Error Bar = SEM.

We next infected neurons with knockdown-replacement lentiviruses expressing YFP-myc-tagged GluK2-WT or GluK2-C2A at DIV 14–15. Seven days later, neurons were treated with 2 μM tetrodotoxin (TTX) and 40 μM GYKI53655 for 30 min, then stimulated with 10 μM KA for 20 min. Surface expressed proteins were then labelled with membrane impermeant biotin, followed by the isolation of biotinylated proteins with streptavidin beads and Western blotting (Figure 5A). Consistent with the SUMOylation data, quantification of the surface expression of GluK2-C2A showed it was significantly reduced compared to GluK2-WT and was not further reduced upon KA stimulation (Figure 5B).

To further confirm these findings, we used confocal microscopy to compare the surface expression and agonist-induced endocytosis of YFP-myc-tagged GluK2-WT and non-palmitoylatable GluK2-C2A in cultured rat hippocampal neurons. Neurons were transfected at DIV 9–10 and at DIV 14–15 treated with 2 μM tetrodotoxin (TTX) and 40 μM GYKI53655 for 30 min to prevent spontaneous synaptic activity, before stimulation with 10 μM KA (20 min) to induce receptor internalisation (Martin et al., 2007a; Konopacki et al., 2011). Neurons were then live labelled with anti-GFP antibodies to measure YFP-myc-GluK2-WT and -C2A surface expression, followed by fixation, permeabilization, and staining to assess total YFP-myc-GluK2 (Figure 5C). Interestingly, and consistent with the surface biotinylation data, the surface expression of GluK2-C2A was significantly reduced compared to GluK2-WT under basal conditions. Moreover, whilst surface expression of GluK2-WT was significantly decreased by KA stimulation (Figure 5D), GluK2-C2A was not, suggesting that preventing GluK2 palmitoylation occludes KA-induced receptor endocytosis.

Taken together, our data support a model whereby activity-dependent depalmitoylation of GluK2 leads to enhanced PKC-mediated phosphorylation at S868, receptor SUMOylation, and KAR endocytosis.

## Discussion

We adapted and optimised an APEGS assay to investigate palmitoylation of the GluK2 KAR subunit and its interplay with other PTMs. The APEGS assay allows us to explore the palmitoylation pattern of low abundance palmitoylated proteins under native conditions with high sensitivity. Previous radiolabelled [^3^H]-palmitate assays in heterologous systems indicated that GluK2 can be palmitoylated at C-terminal cysteine residues C858 and C871. In those studies, both C858A and C871A mutants showed reduced palmitoylation, but it was unclear if both cysteines were simultaneously palmitoylated (Pickering et al., 1995; Copits and Swanson, 2013). We engineered a construct in which both C858 and C871 were mutated to alanine, GluK2-C2A. We show that whilst GluK2-C2A exhibits no palmitoylation, GluK2-WT is palmitoylated at both cysteine residues simultaneously when recombinantly expressed in HEK293T cells, and endogenously in cortical neurons and brain. More importantly, our data indicate that KA stimulation causes depalmitoylation of endogenous GluK2, suggesting that dynamic depalmitoylation may contribute to activity-dependent KAR trafficking.

We are mindful that the KAR stimulation-evoked decrease in GluK2 palmitoylation in neurons is relatively subtle. We observed a statistically significant decrease in doubly palmitoylated GluK2 with an apparent, but not statistically significant, increase in the mono-palmitoylated species. Given this reduction in doubly palmitoylated GluK2 but the continued presence of mono-palmitoylation, it seems likely that only one of the two palmitoylation sites is altered by KAR activation, but further work will be required to test this possibility directly.

**Figure 6:**
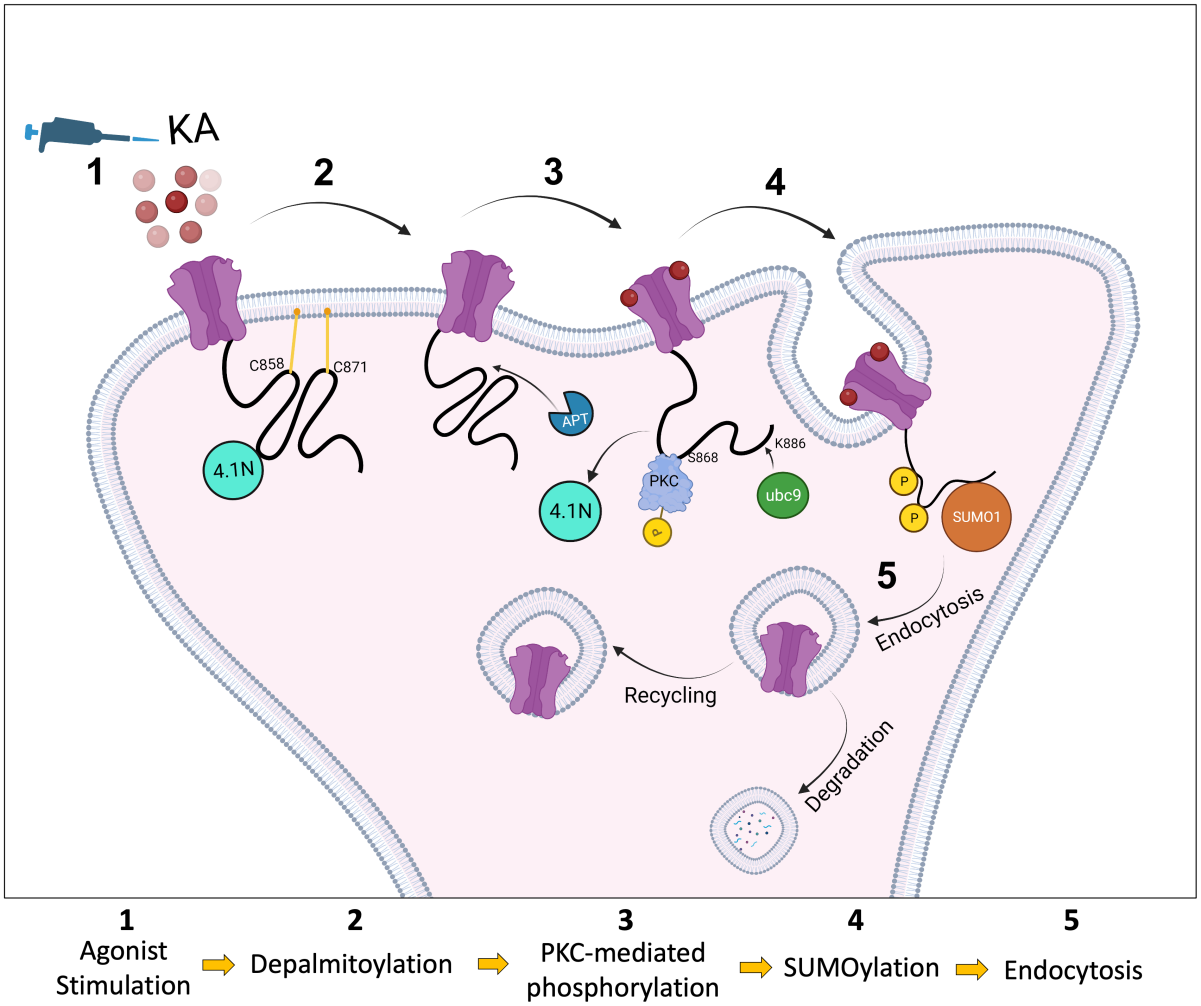
Schematic model. The C-terminus of the KAR subunit GluK2 has two cysteine residues (C858 and C871) that are substrates for palmitoylation (1). Depalmitoylation of GluK2 occurs in response to agonist activation (2) and leads to phosphorylation of the nearby serine, S868, by PKC (3). PKC phosphorylation then promotes SUMO1 conjugation to GluK2 at K886, resulting in receptor internalisation (4). SUMOylated KARs can either be sent back to the surface by recycling or go through the degradation pathway via lysosomes (5).

We used a lentiviral approach to knock down endogenous GluK2 in cortical neurons and replace it with YFP-myc-GluK2-WT or YFP-myc-GluK2-C2A. Using phos-tag electrophoresis we observed that preventing palmitoylation of GluK2 enhanced PKC phosphorylation. An increase in GluK2 phosphorylation of non-palmitoylated GluK2 in heterologous systems has also been reported previously (Pickering et al., 1995), however, it had not been demonstrated in neurons. Here, we extended these findings by showing that preventing palmitoylation of GluK2 specifically enhances phosphorylation of the S868 PKC phosphorylation site, further supporting the proposal that interplay between palmitoylation and phosphorylation acts to fine-tune KAR trafficking. Potentially, depalmitoylation acts to ‘release’ the C-terminal domain of GluK2 from the inner membrane leaflet to allow access for PKC phosphorylation (Figure 6).

Since KA stimulation leads to depalmitoylation of GluK2 and PKC phosphorylation at S868, we examined SUMOylation of non-palmitoylatable GluK2. In our previous studies, we demonstrated that agonist-induced SUMOylation of GluK2 is required for KAR endocytosis. Internalisation assays demonstrated a defect in activity-dependent endocytosis when GluK2 SUMOylation was prevented, and infusion of conjugatable SUMO1 into neurons led to a run-down in KAR currents (Martin et al., 2007a). Subsequently, we observed that phosphorylation of GluK2 at S868 promotes GluK2 SUMOylation and is required for LTD of KAR-mediated transmission at hippocampal mossy fibre synapses (Konopacki et al., 2011; Chamberlain et al., 2012). Consistent with these findings, non-palmitoylatable GluK2 exhibited enhanced SUMOylation, which was not further increased by agonist treatment, suggesting GluK2 depalmitoylation represents a permissive step in mediating receptor phosphorylation, SUMOylation and subsequent endocytosis. In agreement with this model, GluK2-C2A exhibited lower surface expression levels compared to the GluK2-WT under basal conditions and was insensitive to agonist-induced endocytosis. Thus, our data are consistent with activity-dependent interplay between palmitoylation and SUMOylation representing a requirement for agonist-induced GluK2 endocytosis.

The regulation of protein–protein interactions and synaptic vesicle dynamics by the coordinated, often bidirectional, actions of palmitoylation and phosphorylation has been reported to play a role in striatal dopamine release (Charych et al., 2010), neuronal outgrowth (Gauthier-Kemper et al., 2014) and synapsin 1-mediated clustering of synaptic vesicles (Yan et al., 2022). Directly relevant to our work, phosphorylation of GluK2 has been reported to eliminate the interaction of GluK2 with the scaffolding protein 4.1 N, thereby decreasing KAR surface expression. Conversely, the same study reported that GluK2 palmitoylation promotes interaction with 4.1 N, stabilising KARs at the cell surface (Copits and Swanson, 2013). Taken together, our results suggest that activity-dependent depalmitoylation, followed by consequent S868 phosphorylation and SUMOylation, may act collectively to mediate uncoupling of GluK2 from 4.1 N, to facilitate receptor endocytosis in response to agonist activation.

As illustrated in Figure 6, our data indicate that GluK2 subunits of KARs are basally palmitoylated at two cysteine residues. Agonist-induced PKC-mediated phosphorylation of GluK2 acts as an intermediate that ‘links’ depalmitoylation to SUMOylation, and consequent endocytosis. Together, these findings provide a novel viewpoint on the molecular mechanisms by which PTMs cooperate to regulate KAR trafficking and surface expression in response to neuronal activity, and add new insights into the role of palmitoylation in synaptic mechanisms.

## Data availability statement

The raw data supporting the conclusions of this article will be made available by the authors, without undue reservation.

## Ethics statement

All animal procedures relating to this study were approved by the Animal Welfare and Ethics Review Board at the University of Bristol. The study was conducted in accordance with the local legislation and institutional requirements.

## Author contributions

BY: Conceptualization, Formal analysis, Investigation, Methodology, Validation, Visualization, Writing – review & editing. EA: Conceptualization, Formal analysis, Investigation, Methodology, Validation, Writing – review & editing. AE: Conceptualization, Investigation, Writing – review & editing. RS: Writing – review & editing, Methodology, Resources. KW: Investigation, Methodology, Project administration, Resources, Writing – review & editing, Conceptualization, Supervision. JH: Conceptualization, Project administration, Supervision, Funding acquisition, Writing – original draft.
